# Linking inflammatory mediators and indicators of insulin resistance in anthropometry specified type 2 diabetic males

**DOI:** 10.6026/97320630018998

**Published:** 2022-10-31

**Authors:** Venkata Ramesh Bonam, Abu Raghavan Srinivasan, Daniel Devaprasad Manoj

**Affiliations:** 1 Department of Biochemistry, Apollo Institute of Medical Sciences and Research (AIMSR), Chittor campus, Murukambattu, Chittoor 517127 Andhra Pradesh, India; 2 Department of Biochemistry, Mahatma Gandhi Medical College & Research Institute, Sri Balaji Vidyapeeth (SBV),SBV Campus, Pillaiyarkuppam, Pondicherry - 607 402, India; 3 Department of General Medicine, Apollo Institute of Medical Sciences and Research (AIMSR), Chittor campus, Murukambattu, Chittoor 517127 Andhra Pradesh, India

**Keywords:** T2DM, insulin resistance, inflammation, male

## Abstract

Inflammation associated with insulin resistance is a risk factor in the development of complications in Type 2 diabetes mellitus (T2DM). The study was conducted to assess the relationship between inflammatory mediators and insulin resistance, independent of lipid profile in anthropometry specified male Type 2 diabetics. 180 males having T2DM for more than 5yrs and on diabetic medication were chosen for the study and categorized into obese and overweight. Patients with thyroid or other endocrine disorders, kidney, muscle, liver, systemic, and inflammatory diseases were excluded from the study. Blood glucose, glycated hemoglobin, plasma insulin, and the inflammatory biomarkers namely hs-CRP, ferritin, haptoglobin, and adiponectin were evaluated. HOMA-IR and QUICKI were computed to assess insulin resistance. The study demonstrated significant changes in adiponectin and hsCRP in obese and overweight T2DM. However, Ferritin and Haptoglobin were insignificant. The entire biochemical study was carried out to demonstrate lipid profile independent associations. The significant insulin resistance associated with a substantial increase in hs-CRP levels and a pronounced decrease in the adiponectin levels suggests impending diabetic complications in anthropometry specified male T2DM. This could promote the use of personalised medicine to regulate levels of hs-CRP or to improve the secretion of adiponectin thereby countering insulin resistance in T2DM, independent of the lipid profile which is the novelty of our study.

## Background:

An alarming increase in the incidence of Type 2 Diabetes mellitus (T2DM) in developing economies like India poses serious concern. According to International Diabetes Federation (IDF), Southeast Asia is ranked second with 88 million people who are diabetic [1]. T2DM is a progressive, metabolic disorder with micro and macrovascular complications. The prevalence of T2DM is correlated with obesity and unhealthy lifestyles. Several environmental, metabolic and genetic risk factors are associated with T2DM, and obesity is regarded as a cardinal risk factor for T2DM. According to the World obesity atlas 2022, by 2030 approximately 1 billion people globally will be living with obesity [2]. This means that 1 in 5 women and 1 in 7 men will be obese. Obesity is more prevalent among women than men. International classification of disease (ICD) defines obesity as a chronic, relapsing, multifactorial disease. This seems to affect low and middle-income countries (LMIC) where malnutrition continues to be a public health concern2 [World obesity atlas report, 2022]. Obesity is a major risk factor in the progression of T2DMand is characterized by low-grade inflammation in key tissues involved in metabolism. This chronic inflammation especially in adipose tissue and liver is attributed to enhanced nutrient flux which is associated with the progression of insulin resistance (IR) [3]. The present study focuses on the levels of inflammatory mediators in obese and overweight T2DM males and their relationship with insulin resistance. The inflammatory mediators assessed in the present study are ferritin, hs-CRP, haptoglobin, and adiponectin. Furthermore, HOMA-IR (Homeostatic Model Assessment for Insulin Resistance), plasma insulin levels, and glycated hemoglobin (HbA1c) were also investigated to comprehend the association between insulin resistance and inflammation in T2DM. Ferritin is an iron sequestration protein and is a serum biomarker whose elevated levels point to an underlying disease [4]. As this is an acute phase reactant, increased ferritin concentration does not correlate with iron overload under pathological conditions. Recent research has revealed that iron metabolism is closely associated with metabolic syndrome and T2DM.Patients with increased FBG (fasting blood glucose) and diabetic complications such as retinopathy, nephropathy, and vascular dysfunction were found to have elevated serum ferritin levels [5].Pronounced aberrations in the iron homeostasis could result in iron overload and deficiencies. Studies in the Chinese population indicated higher ferritin concentrations in the serum of T2DM patients compared to the control group. Additionally, an increase in the serum ferritin levels in non-diabetic patients is suggestive of impaired glucose tolerance [6]. Hence, the current study has been envisaged to decode the nexus between serum ferritin levels, and T2DM in overweight and obese patients. High sensitivity CRP (hsCRP) is a type of CRP (C-reactive protein) and another acute phase reactant protein that implies low-grade inflammation. A study indicated that elevated CRP in the Asian population including Indians is associated with pre-diabetes and CVD risk factors [7]. Pre-diabetes is known to be associated with low-grade inflammation. Hence serum hs-CRP is considered as an independent prognostic risk factor as it is capable of predicting CVDs and metabolic syndrome. A study by Sridevi et al. suggested that hs-CRP be added as a diagnostic criterion as the levels were elevated in T2DM patients with metabolic syndrome [8]. Another study indicated that there is a relationship between hs-CRP and glycemic control [9]. A study on the Indian population also revealed that there is a correlation between CRP and hyperglycemia [10]. Hyperglycemia and increased serum CRP levels are characteristic features of uncontrolled T2DM. Various studies have documented dyslipidemia, high CRP levels, and low-grade inflammation in T2DM. In T2DM elevated hs-CRP contributes to the progression of cardiovascular and renal diseases [11]. Therefore, the present study is a sincere attempt to understand the relationship between hs-CRP and T2DM in overweight and obese males. Haptoglobin (Hp) is yet another acute phase reactant glycoprotein. The major function of Hp is to bind with an affinity of 1 x 10-15 mol/l to oxygenated, free hemoglobin (Hb). Though hemoglobin is an oxygen carrier, free hemoglobin can cause oxidative tissue damage through the Fenton reaction by aiding the accumulation of hydroxyl radicals [12]. The synthesis of this acute phase protein in the liver is triggered by the pro-inflammatory cytokines, namely IL-1, IL-6, and TNF α. Adipocytes and lung cells also express Hp and there is an upregulation in its expression during inflammation [13]. The facilitation of Hb clearance by haptoglobin is greatly dependent on Hp gene polymorphisms and their structural differences which make it a susceptibility marker in diabetes patients. Hence, evaluating the levels of haptoglobin in overweight and obese T2DM patients is essential in deducing the role of this inflammatory mediator in insulin resistance. Adiponectin, an adipokine is an anti-inflammatory 244-amino acid protein found in the bloodstream. Decreased adiponectin levels in plasmais believed to be associated with obesity-linked complications like type 2 diabetes. Studies suggest that hypoadiponectinemia is observed in obese subjects compared with non-obese subjects. Several studies in the clinical setting also illustrate a connection between hypoadiponectinemia and insulin resistance in type 2 diabetes [14]. The HOMA-IR (Homeostatic Model Assessment for Insulin Resistance) is considered to be a potent tool for the measurement of IR. It is calculated by using the following expression i.e. HOMA-IR = Fasting insulin (mIU/ml) x fasting glucose (mmol/L)/22.5(FPIxFPG)/22.5 [15]. A threshold value of 2.5 is taken as a benchmark for IR in adults [16]. Homeostasis Model Assessment of beta-cell function (HOMA-B) is also performed [17]. HOMA-B is employed to monitor pancreatic beta cell function. Monitoring glycemic control in patients with diabetes mellitus has long been considered as essential. Therefore, glycated hemoglobin (HbA1c) levels are measured. The HbA1c assay is the gold standard and is an accurate, precise measure of chronic glycemic levels. These levels can be correlated with the risk of diabetes complications [18]. Hence, in this study, HbA1C levels are assessed in overweight and obese T2DM patients. Thus, the aimis to unravel the role of inflammatory mediators in the development/ progression of insulin resistance in overweight and obese male type 2 diabetics.

## Subjects:

### Study design:

This study was performed at a tertiary health care set up in South India. The study included a total of 180 T2DM male subjects: 90 overweight subjects and 90 obese subjects. The research study was duly approved by the Research Advisory Committee (RAC) and Institutional Human Ethics Committee (File no IEC15/AIMSR/02/2018). Based on BMI and waist-to-hip ratio, obese and overweight subjects were categorized. Consent from the participants was obtained after describing the purpose of the study in detail. Care was taken to explain in the vernacular language the nuances and benefits of the study and the minimum discomfort that the subjects would experience while giving the blood samples. Appropriately qualified clinicians diagnosed the chosen subjects and their T2DM was confirmed both clinically and biochemically. Subjects with T2DM for more than 5yrs and on diabetic medication were chosen for the study. Patients with thyroid or other endocrine disorders, kidney, muscle, liver, systemic, and inflammatory diseases were excluded from the study [19].

### Inclusion criteria

Overweight and Obese Patients with T2DM of more than five-year duration who were on standard care of treatment (oral hypoglycemic drugs)

### Exclusion criteria

Patients with co-morbidities including thyroid and other endocrine disorders

Patients with kidney, liver and muscle diseases

Patients with inflammatory disorders

## Materials and methods:

Processing of blood samples:The processing of blood samples was essentially based on CLIA guidelines.

## Biochemical assessment:

Fasting blood glucose and postprandial blood glucose were estimated enzymatically (GOD-POD) using an automated procedure; the HPLC method was used for the quantitation of glycated hemoglobin (HbA1C). A quantitative insulin sensitivity check index (QUICKI) was also computed. HOMA - IR (Fasting plasma glucose (mmol/l) x plasma fasting insulin (m IU/ml)/22.5) was used to calculate insulin resistance HOMA-IR was considered high when it is HOMA-IR ≥ 2.69 [20]. HOMA-BETA (β) was also assessed using the formula HOMA-B = 20 x insulin/(glucose − 3.5) [21]. Measurement of inflammatory biomarkers namely ferritin, haptoglobin, adiponectin, and hs-CRP were enabled. Adiponectin and Haptoglobin were quantitated by ELISA, whereas Ferritin and hs-CRP were estimated based on chemiluminescence. The reference ranges for the biochemical parameters are shown in Table 1(see PDF). Stringent Quality control was ensured with reference to biochemical estimations. Internal quality control was in place and External Quality Assessment was enabled through the Clinical Biochemistry Laboratory of CMC Vellore, under the aegis of ACBI.

## Statistical analysis:

ANOVA and linear regression analysis were performed and standard errors were estimated.

## Results:

The biochemical analysis of the study subjects (Male Type 2 DM; obese and overweight) to depict the nexus between inflammatory mediators and indicators of insulin resistance/sensitivity, independent of Lipid profile is represented in the form of a Table (Table 2 - see PDF). The mean age, BMI, and WHR of the patients included in the study were 55.19±1.226 (Obese), 57.44±1.264 (Overweight), 31.939 ±0.2418 (Obese), 27.192 ±0.1567 (Overweight), and 1.02449 ± 0.003611 (Obese), 1.01362 ± 0.003811 (Overweight) (Table 2 - see PDF).

## Glycemic control in obese and overweight T2DM patients:

Glycemic control in overweight and obese T2DM subjects chosen for the study was observed. The mean glycated hemoglobin (HBA1C) was 9.382± 0.2455 in obese T2DM patients while it was 9.373 ± 0.2728 in overweight T2DM patients (Table 2 - see PDF). HbA1C values in the 8-9 range indicate poor glycemic control [22]. The results indicate that obese and overweight male T2DM patients have poor glycemic control.

## Development of insulin resistance (HOMA-IR & QUICKI) in obese and overweight T2DM patients:

Insulin resistance is calculated by HOMA-IR. The HOMA-IR mean value for obese T2DM patients was 5.4405 ± 0.59837 and 3.9205 ± 0.33915 for overweight T2DM patients (Table 2 - see PDF). This data suggests that there is a development of insulin resistance in both obese and overweight T2DM patients. Both subsets have values greater than 2.9, indicating significant insulin resistance (Table 2 - see PDF). Similarly, the QUICKI scores of obese T2DM patients are 0.3100 ± 0.00278 and 0.3195 ± 0.00229 for overweight T2DM patients. As the QUICKI scores below 0.339 indicate insulin resistance (Table 2 - see PDF). Our data reveals that there is substantial insulin resistance observed in both the subgroups.

## Study of beta cell function (HOMA-BETA) in obese and overweight T2DM patients:

The beta cell function was estimated through the HOMA model in all the subjects included in our current study. The HOMA-BETA (β) values were 58.5456±7.15245 for obese subjects and 36.3632±5.51858 for overweight subjects (Table 2 - see PDF). Lower HOMA-BETA levels indicate increased insulin-resistant T2DM as compared to the >150 range [17].

## Ferritin as an inflammatory biomarker in obese and overweight T2DM patients:

Ferritin levels in the serum were assessed for all the 180 overweight and obese T2DM subjects. The serum ferritin levels observed in obese T2DM were 135.0095±15.91298 and 93.6642 ± 7.89395 for overweight T2DM (Table 2 - see PDF). No significant increase in the inflammatory biomarker ferritin was observed in both groups.

## Role of adiponectin in the regulation of inflammation and insulin sensitivity in obese and overweight T2DM patients:

Adiponectin is a peptide hormone that is adipose tissue-derived. The mean adiponectin levels for obese T2DM patients were 0.31249 ± 0.009596 and 0.31608± 0.011550 for overweight T2DM patients (Table 2 - see PDF). The current study indicates that there is a significant decrease in the circulating levels of adiponectin in overweight and obese T2DM subjects. This can be negatively correlated with insulin resistance. Lower levels of adiponectin are a predisposing factor for insulin resistance while higher levels indicate increased insulin sensitivity [14, 23].

## Hs-CRP as an inflammatory biomarker in obese and overweight T2DM patients:

In addition to ferritin, hs-CRP is an acute phase reactant assessed in this study. The mean hs-CRP levels assessed were 4.876 ± 0.3916 for obese T2DM and 3.855 ± 0.3842 for overweight T2DM patients (Table 2 - see PDF). In both subgroups considered for the study, hs-CRP remained significantly higher, indicating systemic inflammation. Elevated hs-CRP is indicators of chronic inflammation and is a risk for subsequent diabetic complications [11].

## Inflammatory marker haptoglobin, an acute phase protein in obese and overweight T2DM patients:

Serum levels of haptoglobin (Hp) in both obese and overweight T2DM subjects continue to remain within the normal range of 0.5-2.2g/dL. The mean serum Hp levels of obese T2DM subjects are 0.2808 ±0.00780 and 0.2775 ± 0.00778 for overweight T2DM subjects (Table 2 - see PDF). This data suggests that there is no significant increase in the Hp levels.

## Discussion:

Research over the decades has illustrated that obesity can initiate inflammation in adipose, skeletal muscle, liver, gut, pancreatic, and brain tissue involving increased polarization of immune cells and release of inflammatory cytokines. This might ultimately contribute to insulin resistance specifically in type 2 diabetes mellitus [24]. Therefore, our study aims to understand the relationship between obesity, T2DM, insulin resistance, and inflammation. From our analysis of 180 subjects, 80 obese T2DM subjects, and 80 overweight T2DM subjects we observed significant insulin resistance in both categories. This was accompanied by an elevation in the levels of inflammatory biomarker hs-CRP and a decline in the levels of the adipose-tissue hormone adiponectin, though the levels of ferritin and haptoglobin remained within the normal range. Increased BMI 31.939 ±0.2418 (Obese), and 27.192 ±0.1567 (Overweight) (Table 2 - see PDF) as observed in this study have always been correlated with decreased insulin sensitivity. Furthermore, in T2DM, obesity and overweight are linked to the worst glycemic control [25]. Studies have revealed that the development of insulin resistance is the result of local and systemic inflammation induced by obesity. Simultaneously, insulin resistance and hyperinsulinemia can also contribute to obesity [26]. Inflammation might play a pivotal role in insulin resistance and is capable of exacerbating each other. In obesity, inflammation begins in the adipose tissue that contributes to insulin resistance within the adipose tissue. This process occurs through the autocrine effects of inflammatory mediators on insulin signalling and metabolism. Furthermore, these can influence the insulin sensitivity of the other tissues. In the case of systemic insulin resistance and T2DM, inflammation accelerates the fat spillover from adipocytes to the liver and skeletal muscle tissue [27-28]. The mean glycated hemoglobin levels of obese and overweight T2DM subjects in the present study was more than 8.0 indicating poor glycemic control. Similarly, insulin resistance assessed with HOMA-IR and QUICKI revealed the development of significant insulin resistance in both obese and overweight T2DM. The mean values of HOMA-IR in both groups were greater than 2.9 indicating grave insulin resistances. The QUICKI values also suggest that there is insulin resistance in both obese and overweight T2DM Obesity, glycemic control, and insulin resistance are three main interlinked factors in T2DM. In coherence with the earlier studies, our study has also observed that poor glycemic control, overweight, and obesity are closely associated.

Impairment of β-cell function is yet another important factor in T2DM. Increased insulin resistance correlates with decreased β- cell. As insulin resistance was evaluated with HOMA-IR, the β-cell dysfunction is evaluated with HOMA-BETA (β). Increasing glucose concentrations in the arteries induce the pancreatic β-cells to secrete insulin. Initial glucose rise in the arteries causes the insulin release which is the first phase and peaks at 2-4 min this then reduces sharply by 10-15 min, followed by a gradual second-phase insulin release at about 2-3h. In the case of insulin resistance, as insulin sensitivity is compromised there is stimulation of β-cells to secrete increased amounts of insulin than under normal conditions [29]. These along with glucolipotoxicity and obesity-related inflammation, culminate in hyperglycemia and, eventually T2DM [30]. Earlier studies indicated that the severe insulin-resistant Indian subgroup had a low β-cell function, but this is not a common characteristic observed. In certain Swedish subgroups, severe insulin-resistant is not accompanied by low β-cell function [17]. Even within the Indian population, this characteristic varies with age, BMI, and genetic predispositions. Similarly in this study with 180 obese and overweight subjects, we found that there was a significant decrease in β-cell function along with an increase in insulin resistance. HOMA-BETA values were 58.5456± 7.15245 for obese and 36.3632±5.51858 for overweight subjects with HOMA-IR as 5.4405 ± 0.59837 and 3.9205 ± 0.33915 demonstrating a low β-cell function and significant insulin resistance (Table 2 - see PDF). An earlier research study suggested insulin resistance that manifests as impaired insulin-stimulated glucose transport and inhibition of lipolysis in adipose tissue. A divergence in insulin signalling in adipocytes is observed wherein its effect on the glucose transporter-4 trafficking is blunted but its effect on Forkhead box O-1 (FoxO1) nuclear exclusion is preserved [31]. In obesity, adipocyte-insulin resistance can be produced through cell-autonomous mechanisms or inflammatory mediators. Obesity-related insulin resistance involves initiating inflammation by releasing pro-inflammatory cytokines, aging, adipocyte dysfunction through hypoxia, oxidative stress, ER stress (endoplasmic reticulum stress), and genetic predisposition. Increased inflammatory cytokines like TNF-α and IL-6 secreted by visceral adipocytes along with cytokines specific for adipocytes are the key players in inducing insulin resistance [32]. In our study with 90 obese T2DM and 90 overweight T2DM patients, we found the inflammatory biomarker hs-CRP levels to be >3 which accounts for high risk (Table 2 - see PDF). Both groups have elevated levels of hs-CRP. This data suggests that there is an association between obesity, overweight, T2DM, and hs-CRP. Several longitudinal studies have correlated the development of insulin resistance to change in hsCRP levels. A longitudinal study that investigated the bidirectional relationship between inflammation and insulin resistance showed that baseline levels of hsCRP and IL-6 (interleukin-6) were positively linked with consequent elevations in fasting insulin, HOMA-IR, and beta-cell function. The reverse does not occur [33]. This further validates our study wherein insulin resistance and increased hs-CRP levels were observed in both obese and overweight T2DM subjects.

Adiponectin plays a crucial role in various metabolic and cellular processes and is a widely accepted biomarker for obesity-related metabolic diseases including T2DM, and, CVD. As adiponectin has diverse roles, BMI and serum adiponectin levels have an inverse correlation. Hypoadiponectinemia and insulin resistance have a positive correlation, studies have illustrated decreased adiponectin levels in patients with insulin resistance despite obesity [34]. Obesity-associated metabolic syndrome and insulin resistance are primarily due to adipokine dysregulation. In the current study, we have observed that obese and overweight T2DM subjects with a mean BMI of 31.939 ±0.2418 (Obese), and 27.192 ±0.1567 (Overweight) had very low levels of serum adiponectin (Table 2 - see PDF).

The mean serum adiponectin levels were 0.31249 ± 0.009596 and 0.31608± 0.011550 (Table 2 - see PDF) in obese and overweight T2DM patients respectively. This data is indicative of the fact that there is an inverse correlation between BMI and serum adiponectin. Furthermore, as previous research as demonstrated a positive correlation between insulin resistance and lower serum adiponectin levels the present study also expresses the same inference [35]. This study data validates that a significant increase in insulin resistance is accompanied by a decline in the levels of serum adiponectin. The role of adiponectin in insulin resistance, obesity, and inflammation is yet to be clearly delineated. But it elicits a series of downstream signalling events that make it an insulin sensitizer via its molecular cross-talk with the insulin signalling pathway. PI3K/AKT signalling pathway is the main metabolic pathway for insulin to carry out its essential functions such as protein synthesis, lipogenesis, utilization and uptake of glucose, glycogen synthesis, and reduced lipolysis and gluconeogenesis. Adiponectin and its receptor interactions (Adipo R1 and R2) result in the activation of various signalling pathways like p38 MAPK AMPK and IRS1/2. Insulin sensitization by adiponectin mainly involves IRS1/2 by signalling in insulin-responsive tissues [36]. Therefore, this study establishes the association between serum adiponectin levels, BMI, insulin resistance, and inflammation.

## Conclusions:

The present study has enabled us to validate the association between hs-CRP and adiponectin in insulin resistance. We found that BMI, elevated hs-CRP, decreased serum adiponectin and insulin resistance have a cascading link that are interdependent, but acquire relevance independent of the status exhibited by the Lipid Profile. These metabolically linked factors pave way for the underlying diabetic complications and CVD risk in obese and overweight male T2DM.

## Novelty of the study (novel hypothesis):

Our study has strived to decipher the nexus among indicators of insulin resistance, hs-CRP, and, adiponectin in anthropometry specified type 2 male diabetics. The novelty of the study is fortified further by the fact that the relationship has been studied independent of lipid profile. We had hypothesised that insulin resistance would be linked to inflammatory mediators in anthropometry specified male type 2 diabetics, independent of lipid levels and proved the same. The study could thus aid in personalised medicine/precision medicine to help overcome systemic inflammation in regulating adiponectin thereby countering insulin resistance and delaying the onset of complications in T2DM, independent of lipid profile.

## Limitations of the study:

Female type 2 diabetics did not constitute the study subjects. Haptoglobin and Adiponectin SNPs were not studied.

## Scope for future studies:

It would be worth carrying out studies on the probable association featuring hs-CRP and adiponectin gene polymorphism, namely SNP+45, SNP+276, and elicit characteristic findings in obese and overweight type 2 diabetics, irrespective of gender. It is also worth studying haptoglobin polymorphism in the light of the above. This might open newer vistas in molecular medicine and biochemical pharmacology aimed at T2DM.
